# Case Report: Repeated Low-Dose Rituximab Treatment Is Effective in Relapsing Neuro Behçet's Disease

**DOI:** 10.3389/fneur.2021.595984

**Published:** 2021-04-15

**Authors:** Chao Zhao, Chuan Li, Feng-ju Duan, Qi Yan, Zhuo Zhang, Ying Du, Wei Zhang

**Affiliations:** Department of Neurology, Tangdu Hospital, Fourth Military Medical University, Xi'an, China

**Keywords:** neuro Behçet disease, treatment, rituximab, low dose, remission

## Abstract

Neuro Behçet's disease (NBD) is a rare but most aggressive manifestation of Behçet's disease (BD) with a poor prognosis, and some patients even present a relapsing and treatment-resistant progressive course. In some relapsing NBD cases, traditional corticosteroids and immunosuppressive drugs show limited efficacy, while benefits of biological agents, such as anti-B-lymphocyte CD20 biological agent rituximab (RTX), gradually represent potential therapeutic advantages with clinical rapid remission and long-time maintenance. However, up to now, the optimal dosage of RTX in NBD is still elucidated. Here, we report two patients with relapsing NBD, despite continuous high dose steroids and sufficient azathioprine treatment, still presenting severe and relapsing meningoencephalitis or brainstem involvement. Repeated low-dose RTX (100 mg × 3/1 week apart, 100 mg repeated every 6 months) is then attempted with rapid recovery and sustained remission. The approach in our cases may expand therapeutic options and provide helpful references for relapsing NBD treatment.

## Background

Behçet's disease (BD), a chronic and relapsing multisystem disorder, is an auto-inflammatory-caused vasculitis, leading to venous or arterial thrombosis, oral or genital aphthosis, uveitis, polyarthritis, and even rare gastrointestinal, cardiac, pulmonary, or neurological manifestations. Among them, neurological involvement of BD, also called neuro Behçet's disease (NBD), is one of the most aggressive manifestations of BD with a poor prognosis. Epidemiological studies in different countries have reported that the estimated prevalence of BD is 0.64–420/10^5^, and the frequency of NBD among BD is ~9% (ranging from 3 to 30%). Compared with the rare involvement of the peripheral nervous system in NBD, the central nervous system is much more vulnerable, and it has been also divided into two forms: parenchymal and non-parenchymal manifestations (1, 2). Parenchymal lesions include meningoencephalitis, brainstem, hemispheric, and spinal cord involvement, while non-parenchymal ones comprise dural sinus thrombosis, arterial occlusion, and aneurysms (3).

The diagnosis of NBD, in the absence of characteristic biological tests, is mainly performed by clinical means as described in International Consensus Recommendation (ICR) criteria. In the definite NBD, patients must be firstly satisfied with International Study Group (ISG) criteria for BD, and they must then present neurologic symptoms and/or signs caused by BD, be supported by relevant abnormalities in neuroimaging and/or CSF, and have any other neurological diseases be excluded (4). In general, 30% of neurological lesions in NBD respond well to traditional corticosteroids and immunosuppressive agents with a monophasic process, whilst the remainder frequently relapse and gradually develop a treatment-resistant progressive course (5). Therefore, additional therapies for a subset of relapsing patients with NBD are inevitably needed. Indeed, accompanied by progression in the pathogenetic mechanisms of NBD, the treatments have been revolutionized by biological agents such as anti-cytokines and anti-B-lymphocytes (6).

In clinical practice, the biological agent monoclonal antibody rituximab (RTX), specifically targeting B-lymphocyte differentiation membrane antigen CD20, has been increasingly used in recent years for some immune-mediated diseases, and it has gradually represented potential therapeutic advantages with rapid recovery and sustained remission in relapsing NBD. However, up to now, empirical RTX off-label use in NBD is intravenously administrated by reference to high-dose in the conventional treatment of lymphomas (375 mg/m^2^/week for four cycles) and rheumatoid arthritis (1,000 mg × 2/2 weeks apart, repeated every 6 months), exerting more medical expenses and adverse events (7–9). Therefore, the optimal dosage of RTX for both safety and efficacy in NBD needs to be explored and elucidated. Here, we report two relapsing NBD cases, despite continuous high-dose steroids and sufficient azathioprine treatment, that still present severe and relapsing meningoencephalitis or brainstem involvement. Repeated 100 mg low-dose RTX is attempted with immediate significant improvement and continuous lack of recurrence. The approach of repeated low-dose RTX in our cases may expend therapeutic options and provide helpful references for relapsing NBD treatment.

## Case Presentation

Case 1 involved a 49-year-old Chinese male who complained of progressive vision loss in the right eye, slurred speech, and left limb weakness for 3 days before being hospitalized in our department. The patient suffered from bouts of multiple oral and genital ulcerations from age 46, and he gradually developed recurrent arthritis in the left elbow and occasional skin nodular rash in the trunk. At age 48, he experienced sudden vertigo, nausea, and vomiting followed by continuous dizziness and both lower limbs weakness. An MRI showed multiple lesions in the brainstem, presenting a high signal in T2-weighted sequences, and abnormalities in the CSF were elevated protein levels and white cell count (data not available). Based on these symptoms and examinations, the patient was then diagnosed with neuro Behçet's disease (NBD) at a local hospital. The treatment of intravenous methylprednisolone 1,000 mg/day for 5 days was administrated with rapid improvement after 1 week, and this was followed by high-dose prednisone 60 mg/d and 100 mg azathioprine, gradually adding to 200 mg per day for maintenance (about 3 mg/kg/day), which is adequate for recommended dosage of azathioprine 2–3 mg/kg/day in NBD (6). A total of 9 months later, the patient barely tolerated the side effects of prednisone and quickly withdrew after 1 month. Despite a sufficient dose of azathioprine 200 mg per day, the patient still relapsed with confusion and slurred speech after 2 months and was transferred to our department.

On admission, physical examinations showed oral and genital ulcerations without a skin rash, a swollen right optic disc with only light perception, and branch retinal vein occlusion on the same side without uveitis. Neurological examination showed drowsiness, decreased attention, dysarthria, absence of pharyngeal reflex, left-side ataxia, and hypoesthesia of the right face and left trunk without meningeal irritation signs. Proximal and distal muscle strength of left limbs were 4/5 by manual muscles testing (MTT) accompanied by brisk left tendon reflexes and left Babinski sign. No positive family history of neurological disease had been recorded before.

Erythrocyte sedimentation rate and C-reactive protein (CRP) were 30 mm/h (normal <15 mm/h for male) and 25.6 mg/L (normal <3 mg/L), respectively. The spectrums of autoantibodies, rheumatoid factor, anti-CCP antibody, and ANCA were all negative. The values for various routine and biochemical examinations were within their normal ranges. Tests for infection, HLA-B27, and HLA-B51 were also negative. A lumbar puncture showed a normal opening pressure, and biochemical and cytologic tests of CSF revealed elevated protein level 0.82 g/L (normal 0.15–0.45 g/L), increased white cell count 76 × 10^6^/L (normal <5 × 10^6^/L), and a composition that was 77.8% lymphocyte with normal glucose and chloride. CSF cultures and tests for infection, NMOSD including anti-aquaporin-4(AQP4) antibody and anti-myelin oligodendrocyte glycoprotein (MOG) antibody, MS (oligoclonal bands), autoimmune encephalitis, and paraneoplastic antibodies were all negative.

**Figure 1:**
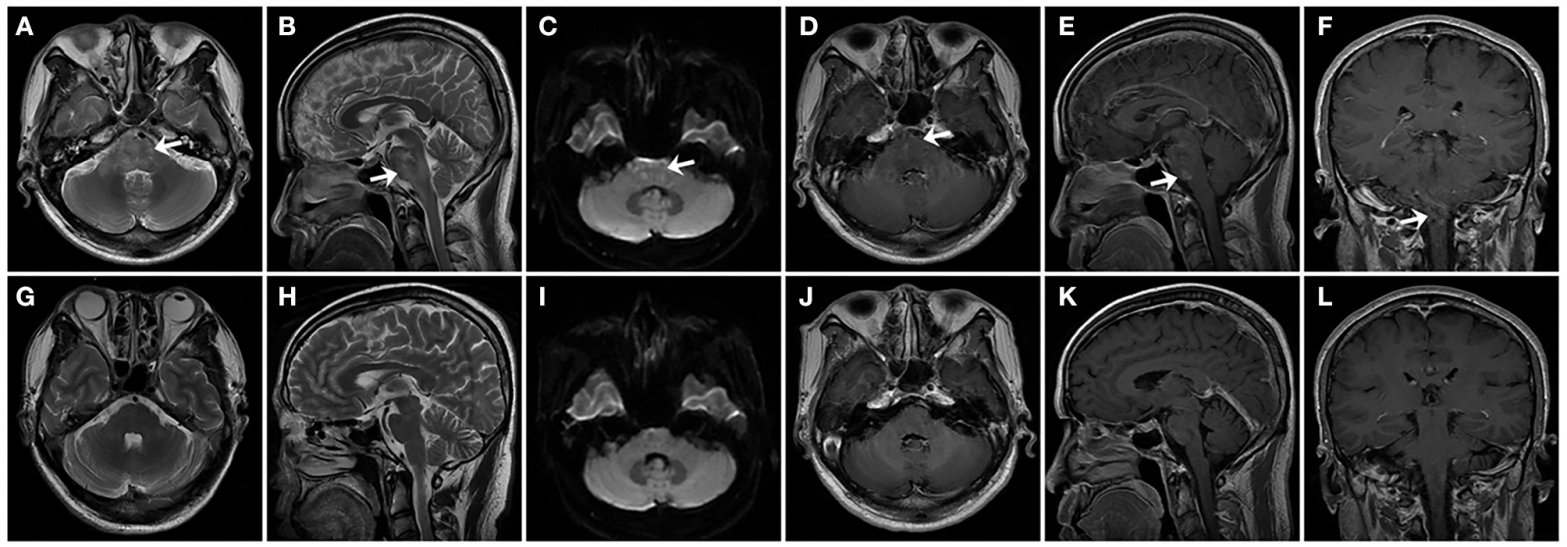
In patient 1, series of brain MRI revealed small and scattered lesions distributed in the pons (indicated by arrows), presenting hyperintensity on horizontal and sagittal T2-weighted images **(A,B)**, high signal on the diffusion-weighted image (DWI) **(C)**, and slim contrast enhancement after Gd-DTPA injection on horizontal, sagittal, and coronal position **(D–F)**. After treatment for 6 months, follow-up MRI images were seen in the patient. Horizontal and sagittal T2-weighted images **(G,H)**, DWI **(I)**, Gd-DTPA enhancement on horizontal, sagittal, and coronal position **(J–L)**, showing complete regression of lesions in the pons.

**Figure 2:**
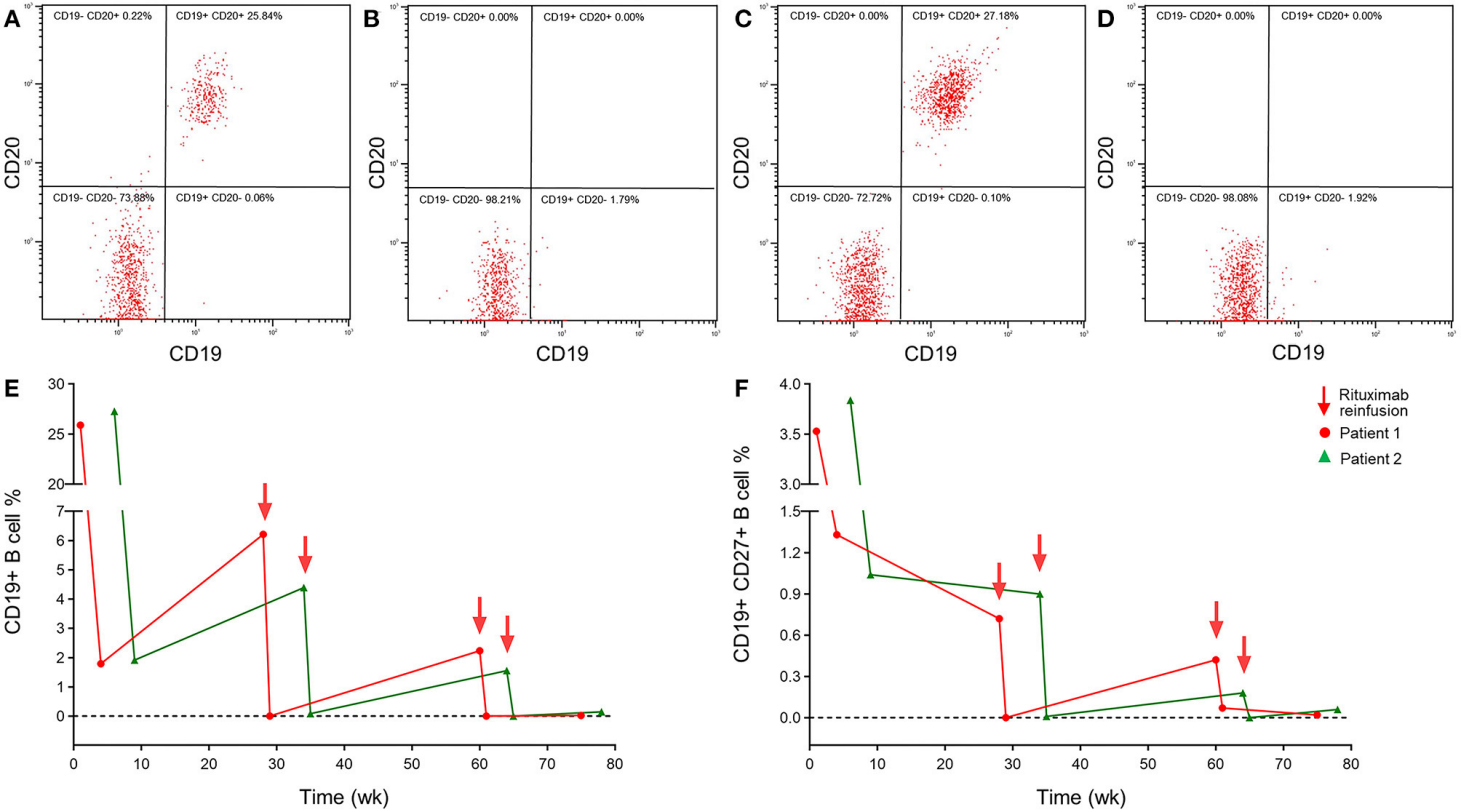
A peripheral blood flow cytometry immunophenotype showed depletion of CD20+ B lymphocytes in NBD patients with reduced-dosage rituximab treatment 100 mg IV once per week for 3 consecutive weeks. Peripheral blood flow cytometry immunophenotype in patient 1 before **(A)** and after **(B)** 3 weeks treatment, patient 2 before **(C)**, and after **(D)** 6 weeks treatment. **(E)** The repopulation of CD19+ B lymphocytes in patients with NBD during rituximab therapy still remains at a lower level. **(F)** The lower repopulation of CD19+CD27+ B lymphocytes paralleled that of CD19+ B cells after rituximab treatment.

A series of brain MRIs revealed small and scattered lesions distributed in the pons, presenting hypointensity on T1-weighted, hyperintensity on T2-weighted images, high signals on the diffusion-weighted image (DWI), and slim contrast enhancement after Gd-DTPA injection (Figures 1A–F). Furthermore, the flow cytometry immunophenotype showed a normal percentage of CD3+ total T lymphocyte of 68.3% (normal 59.4–84.6%), an elevated percentage of CD20+ B lymphocyte 26.06% (Figure 2A) (normal 6–20%), a percentage of CD19+ B lymphocytes of 25.90%, and a percentage of CD19+CD27+ B lymphocytes of 3.53% in peripheral blood.

Based on clinical examinations, the diagnosis of NBD was made in the patient. Since the patient refused a continuous immunosuppressive regimen, infliximab and adalimumab being unavailable in our hospital, from the fourth hospital day, he was treated with repeated intravenous low-dose 100 mg RTX once a week for three cycles, with oral prednisolone 30 mg and azathioprine 200 mg per day. The neurological manifestations, such as dysarthria, ataxia, crossed hypoesthesia, and left hemiplegia, were completely resolved, and the visual acuity in the right eye was also improved to 0.1 within 3 weeks. Erythrocyte sedimentation rate and CRP test, CSF protein, and white cell counts were all normal, and the peripheral blood flow cytometry immunophenotype showed a CD20+ B lymphocyte count that had been decreased to 0.00% (Figure 2B), a CD19+ B lymphocyte count of 1.79%, and a CD19+CD27+ B lymphocyte count of 1.33%. After discharge, oral 30 mg prednisone and 200 mg azathioprine were tapering 5 and 50 mg every 2 weeks, respectively, until withdrawal within 2–3 months, and repeated 100 mg RTX was administered every 6 months without relapse during the 1-year follow-up. A total of 6 months later, follow-up MRI images in the patient have shown complete regression of lesions in the pons (Figures 1G–L). The consecutive peripheral blood flow cytometry immunophenotype showed that the percentage of CD19+ ( ≤ 1%) and CD19+CD27+ B cells (≤ 0.05%) still remained at low levels (Figures 2E,F).

Case 2 involved a 34-year-old Chinese female who was admitted to our department with complaints of an intermittent headache and fever for 1 week. The patient suffered from bouts of multiple oral and genital ulcerations and gradually developed recurrent generalized arthralgia of the wrists, shoulders, and knees over the previous 2 years that was occasionally accompanied by a nodular rash on the lower limbs. Her initial investigations showed an erythrocyte sedimentation rate (ESR) of 41 mm/h (normal <20 mm/h), and other laboratory tests were unremarkable. Based on clinical presentation and investigation, the patient was diagnosed with Behçet's disease (BD) 9 months later at a local hospital, started on 40 mg of oral prednisone with great improvement, and tapered slowly to withdrawal in 3 months.

A total of 6 months later, the patient had a symptomatic relapse with significant worsening of oral and genital ulcerations, arthralgia, and the development of an insidious headache with nausea and vomiting. She was then treated with intravenous methylprednisolone 1,000 mg/day for 5 days followed by oral 60 mg prednisone slowly tapering to 5 mg every month and sustained 200 mg azathioprine (about 3 mg/kg/day), which is adequate for recommended dosage of azathioprine 2–3 mg/kg/day in NBD (6), and the condition improved quickly. When oral prednisone was tapered to 30 mg/day in the next 6 months, despite oral azathioprine 200 mg per day, the patient still relapsed and was transferred to our department.

On admission, the patient complained of insidious migraine-like headache with nausea, vomiting, tiredness, and slight fever. Physical examinations showed oral and genital ulcerations, a body temperature 37.3°C, spontaneously generalized joint pain, and tenderness without skin rash; a neurological examination disclosed positive meningeal irritation sign of neck stiffness and brisk tendon reflexes. Mental, cranial nerves, motor, and sensory functions were all normal. No family history of neurological disease and metabolic-related disorders had been previously documented according to her medical records.

Blood analysis showed ESR of 34 mm/h (normal <20 mm/h for female) and CRP of 55.1 mg/L (normal <3.0 mg/L). Autoantibodies to ANA, double-stranded DNA, Sm, Ro, La, Scl-7, Jo-1, ACA, ribonucleoprotein, nucleosomes, and histones were negative. Rheumatoid factor, anti-CCP antibody, and ANCA were also negative. The values for blood glucose, liver function, renal function, electrolytes, serum folate, vitamin B12, homocysteine, lactic acid, tumor markers, thyroid function, and associated antibodies, IL-6, C3, and C4 were in their normal ranges. Tests for bacteria, virus, syphilis, and HIV were all negative. Human leukocyte antigen (HLA) typing for HLA-B27 and HLA-B51 were both negative.

A lumbar puncture was performed and showed a normal opening pressure; the biochemical and cytologic tests of the cerebrospinal fluid (CSF) revealed a normal glucose and chloride level; protein and white cell count were elevated to 0.76 g/L (normal 0.15–0.45 g/L) and 24 × 10^6^/L (normal <5 × 10^6^/L), respectively, with an increasing percentage of lymphocyte 82.6%; CSF cultures and tests for bacteria, viruses, fungi, syphilis, NMOSD (anti-AQP4 and anti-MOG antibody), MS (oligoclonal bands), and autoimmune and paraneoplastic encephalitis were all negative.

A series of brain magnetic resonance imaging (MRI), including T1, T2, diffusion-weight, FLAIR, enhancement, angiography, and venography, were all normal. Moreover, the peripheral blood flow cytometry immunophenotype showed a normal percentage of CD3+ total T lymphocyte 68% (normal 59.4–84.6%) and elevated CD20+ B lymphocyte of 27.18% (normal 6–20%) (Figure 2C), CD19+ B lymphocyte of 27.28%, and CD19+CD27+ B lymphocyte of 3.84%.

Based on clinical examinations, the patient was diagnosed with NBD. Since the patient refused a continuous immunosuppressive regimen, and infliximab and adalimumab were unavailable in our hospital, from the third hospital day, repeated low-dose 100 mg RTX was intravenously administered once a week for three cycles without adjustment of oral prednisone and azathioprine dosage. Her symptoms of headache, arthralgia, and oral and genital ulcerations completely recovered within 3 weeks. Tests of ESR, CRP, CSF protein, and white cell count were all normal, and peripheral blood flow cytometry immunophenotype showed a decreased CD20+ B lymphocyte count of 0.00% (Figure 2D), CD19+ B lymphocyte of 1.92%, and CD19+CD27+ B lymphocyte of 1.07%. The patient was discharged and followed up by oral 30 mg prednisone and 200 mg azathioprine with tapering 5 and 50 mg every 2 weeks respectively until withdrawal in 2–3 months. Repeated 100 mg RTX was administered every 6 months without relapse during the 1-year follow-up so far. Consecutive peripheral blood flow cytometry immunophenotype showed that the percentage of CD19+ B cells (≤ 1%) and CD19+CD27+ B cells (≤ 0.05%) still remained at the low level (Figures 2E,F).

Moreover, both patients were satisfied with the therapeutic effect and achieved continuous remission in clinical and neuroimaging presentation with normal ESR, CRP, CSF protein, and white cell counts during the 1-year follow-up. After regular retreatment without side-effects, by reference to a lower circulating B-cell repopulation (CD19+ B-cell ≤ 1% or CD19+CD27+ B-cell ≤ 0.05%), we planned to prolong the interval period of reinfusion as much as possible in order to gradually taper RTX. However, further studies are warranted to identify the exact regimen for RTX withdrawal.

## Discussion

The exact causes of Behçet's disease (BD) are still unknown, and current knowledge suggested that BD could follow an autoimmune process induced by an infectious or environmental agent in genetically predisposed individuals. The major pathogenetic mechanisms underlying BD were linked to hyperactivity of neutrophils, gammadelta T (γδ T) lymphocytes, Th1 and Th17 lymphocytes, and down-regulation of regulatory T lymphocytes (Tregs) in the innate immunity system, and these have caused critical overproduction of proinflammatory cytokines, such as TNF-α, IL-1, IL-6, and IL-17 (10). However, growing evidence has suggested that B cells might also play a more eminent role in BD, which was not only responsible for antibody production but also involved in antigen presentation, pro-inflammatory cytokine secretion, and modulation of T cell activation. A recent study for peripheral B cell in BD patients without immunosuppressive treatment has reported that reduced circulating B cells numbers reflect a skewing toward the site of inflammation, and this returned to a normal level after treatment (11), which is consistent with our patients for high dose immunosuppressive agents more than 1 year. The development of B cells experienced several stages with corresponding changes in expression of a wide range of cell surface antigens, and CD20 was commonly considered as a specific B lymphocyte marker expressed during maturation from pre-B cells to plasmablasts (12). Moreover, recent researches have found that CD20 was also expressed in a few of the circulating human T lymphocytes by transcription from their own or intercellular transfer from B cells, and these CD20+ T cells presented a strong propensity to cytokine production (13).

The autoimmune reactions in BD were proposed to target primarily blood vessels, especially endothelial cells, resulting in the clinical presentation of vasculitis. Both arteries and veins of any size were involved in BD, including lesions presenting significant neutrophil, mononuclear, and lymphocytic infiltration with immunoglobulin and complement deposition, endothelial cell swelling, and fibrinoid necrosis in histopathology (14). In addition to similar pathological changes, such as BD, destruction of brain tissue with neuronal necrosis, and apoptosis, gliosis and edema have been seen in NBD, most frequently involving the mesodiencephalic junction, cerebellar peduncles, and basal ganglia, as confirmed by MRI (6, 15). Biopsies of NBD lesions revealed striking perivascular cuffs of well-differentiated lymphocytes around blood vessels with focal reactive germinal center formation, and the inflammatory infiltrates were confined to the Virchow-Robin spaces with involvement of vessel walls. Further immunocytochemistry showed these infiltrating cells mostly consisted of CD45RO+ T lymphocytes, CD68+ monocytes/macrophages, and CD20+ B lymphocytes (8, 16).

The disease courses of NBD were different: around a third of patients had single episodes, a third presented repeated relapses, and the other third underwent progressive processes. In a series of retrospective studies, the prognosis of NBD has been reported to be dismal, with residual neurological impairments in about 20–30% of patients and 10-year mortality of 10%, and these rates rose even higher to 65.4 and 35.2% in chronic progress (17, 18). Adverse prognostic predictors were categorized into clinical, CSF, and MRI factors. Among them, clinical presentations of primary progressive course, more than two attacks per year, immediate relapse after corticosteroid withdrawal, brainstem symptoms and paresis, increased cellularity and protein in CSF, or brainstem involvement in MRI at the time of NBD were considered to be signs of poor outcome (6, 19). Therefore, halting progression within a short time and preventing relapse for a long time are the main purposes of dealing with NBD.

In the past, corticosteroids and various immunosuppressive drugs such as azathioprine, mycophenolate mofetil, methotrexate, chlorambucil, and cyclophosphamide (4), were the only available therapeutic options for BD, which often resulted in unstable and incomplete remission and several side effects. However, accompanied by an improved understanding of the molecular mechanisms in BD, novel biological drugs have been developed recently, targeting various cytokine inhibitions or B cell depletion, and they have opened up new and interesting horizons for therapy (20). In a recent review about NBD diagnosis and treatment, immunosuppressive drugs (glucocorticoids, azathioprine, mycophenolate, and cyclophosphamide), as well as biological agents (infliximab or adalimumab for anti-TNF-α, tocilizumab for anti-IL-6, and rituximab for anti-CD20) were all recommended for NBD treatment as Class IIa and Level C (6). Treatments of NBD were encompassed in the BD general protocol except for cyclosporine, which has been reported to be associated with acute neurological damages. Moreover, not all patients were definitely responsible for anti-cytokine agents, and their previous beneficial responses might drop off over time as described in some refractory NBD patients treated with TNF-α antagonists, while B lymphocytes depletion by biologic agent rituximab (RTX) has represented potential therapeutic advantages with rapid recovery and sustained remission in these cases (6–8).

Rituximab (RTX) is a human/murine chimeric monoclonal antibody with a specific affinity for CD20, a differentiation transmembrane protein participating in B lymphocytes activation, and proliferation. RTX has been approved for use in lymphomas and rheumatoid arthritis, and it has also recently increased off-label usage for some immune-mediated diseases such as BD (21). After binding CD20 on the surface of B cells, RTX triggers multiple cell death pathways, resulting in target B lymphocytes depletion, which alters all aspects of B cell participation in the immune response, including antigen presentation, antibody and cytokine production, and indirect effects on T lymphocyte activation/co-stimulation status and functions (22). Moreover, increased CD20-expressing T cells in the blood, which are expected to present both cytotoxic and regulatory features, and have also been directly depleted by RTX (13). These mechanisms of RTX may contribute to the therapeutic effects of autoimmune diseases.

However, the optimal dosage of RTX for both safety and efficacy is still elucidated in NBD, and empirical off-label usage by reference to high-dose lymphomas may lead to more medical expenses and adverse events (23). Indeed, the dysfunction of B cells usually presents with a normal count in autoimmune diseases, which is different from the high tumor burden in lymphomas, and a smaller dose RTX may be sufficient and effective in treatment (24). Recently, reduced low-dose of 100 mg RTX has been tried in some autoimmune disorders, such as neuromyelitis optica spectrum disorders (NMOSD), autoimmune hemolytic anemia (AIHA), and immune thrombocytopenic purpura (ITP), with intravenous administration of one infusion per week for 3 consecutive weeks, and this is repeated according to the percentage of circulating CD19+ B-cell counts. The approach still presented good responsiveness in depleting B cells, improving clinical symptoms, and preventing relapses with favorable side-effects and medical costs (24–26). Moreover, efficacy and duration of RTX therapy in different autoimmune disorders associated with depletion of B cells have been monitored *via* kinetics of the B-cell population, mainly presenting the percentage of CD19+ or CD19+CD27+ memory B cells in peripheral blood by flow cytometry analysis, respectively (22, 24). To the best of our knowledge, there is still a lack of exact analysis of CSF flowcytometry for lymphocytes in NBD up to now, and we regret that we did not perform CSF flowcytometry in our patients. Since BD is mainly an auto-inflammatory-caused vasculitis, which leads to chronic and relapsing multisystem disorder, we only focus on peripheral circulating B-cell counts, which presented limitations in our report. However, as described in a previous study about MS, RTX has been reported to result in the depletion of B lymphocytes both in peripheral blood and CSF (27). Thus, further studies about CSF flowcytometry are warranted in future research of NBD.

In our cases, two patients have been diagnosed as definite NBD according to recent ICR criteria (4), presenting brainstem syndrome of parenchymal subtype in patient 1 and acute meningeal syndrome of non-parenchymal subtype in patient 2. Despite continuously sufficient steroids and azathioprine treatment, the frequent relapses, progressive symptoms, abnormality in CSF, and neuroimaging have always suggested adverse prognosis of our NBD patients. These patients refused the continuous immunosuppressive regimen, and other biological agents, such as infliximab, adalimumab, and tocilizumab, were unavailable in our hospital. Moreover, with the development in the underlying mechanism of hyperactivity and dysfunction of B lymphocytes, and with reference to some refractory NBD and other neurological autoimmune disease cases reported previously (7–9, 24), we firstly chose reduced low-dose 100 mg RTX for these NBD patients with one infusion per week for 3 consecutive weeks, and we found great improvement with depletion of CD20+ B cells. Furthermore, steroids and azathioprine gradually tapered off until withdrawal after 2–3 months of follow-up, and only a repeated low-dose RTX was administrated every 6 months without relapse so far, maintaining a lower percentage of CD19+ and CD19+CD27+ B cells in circulating blood monitored by flow cytometry. Our regimen of low-dose 100 mg RTX seemed not only to present a good response in preventing clinical relapse but was also sufficient for depleting B cells and maintaining a lower level, as previously described in NMOSD (24). To the best of our knowledge, the cases we described here are the first reports of repeated low-dose 100 mg RTX in relapsing NBD treatment. Our report is still limited by its small size, retrospective observation, and short follow-up time, and further studies are warranted to identify the exact regimen for RTX treatment in NBD.

## Conclusion

In summary, based on a series of developments of underlying immune mechanism in NBD, treatment with repeated low-dose 100 mg RTX caused depletion of CD20+ B lymphocytes and may lead to great improvement within a short duration and sustained remission for a long-time in relapsing NBD. Our approach may expand therapeutic options and provide helpful references for relapsing NBD treatment.

## Data Availability Statement

The raw data supporting the conclusions of this article will be made available by the authors, without undue reservation.

## Ethics Statement

Written informed consent was obtained from the individual(s) for the publication of any potentially identifiable images or data included in this article.

## Author Contributions

WZ and YD: study concept and design. CZ, CL, and F-jD: acquisition of data. QY: CSF analysis. ZZ: flow cytometry analysis. All authors: read and approved the final manuscript.

## Conflict of Interest

The authors declare that the research was conducted in the absence of any commercial or financial relationships that could be construed as a potential conflict of interest.

## Abbreviations

BD: Behçet's disease
NBD: neuro Behçet's disease
RTX: rituximab
CSF: Cerebrospinal fluid
MRI: Magnetic resonance imaging
ICR: International Consensus Recommendation
ESR: erythrocyte sedimentation rate
CRP: C-reactive protein
NMOSD: neuromyelitis optica spectrum disorders
MS: multiple sclerosis
TNF-α: tumor necrosis factor alpha
IL: interleukin
γδ T: gammadelta T lymphocytes
Tregs: regulatory T lymphocytes
AQP4: aquaporin-4
MOG: myelin oligodendrocyte glycoprotein.
